# Differential expression of Semaphorin-7A /CD163-positive macrophages in large artery and cardiogenic stroke

**DOI:** 10.1186/s12883-024-03559-6

**Published:** 2024-02-19

**Authors:** Yi Jiang, Zhichao Sun, Zhonglin Ge, Zhonghai Tao, Mengqian Liu, Wen Zhong, Nan Dong, Lei Xu, Hui Wang, Yiwen Xu, Xiaozhu Shen

**Affiliations:** 1 Department of Geriatrics, Bengbu Medical College Clinical College of Lianyungang Second People’s Hospital, Lianyungang, 222000 China; 2 Department of Pathology, Lianyungang Second People’s Hospital, Lianyungang, 222000 China; 3 Department of Neurology, Lianyungang Second People’s Hospital, Lianyungang, 222000 China; 4 Department of Neurology, Shaoxing Central Hospital, Shaoxing, China; 5 Department of Neurology, The First Affiliated Hospital of Soochow University, Suzhou, China; 6 Department of Infectious Disease, The First Affiliated Hospital of Ningbo University, Ningbo, China; 7 Department of Geriatrics, Lianyungang Hospital Affiliated to Jiangsu University, Lianyungang, 222000 China

**Keywords:** Stroke, Thrombus, Sema7A, CD163, Immunohistochemistry

## Abstract

**Background:**

Identification of the causes of stroke of undetermined etiology, specifically cardioembolism (CE) and non-CE causes, can inform treatment planning and prognosis prediction. The objective of this study was to analyze the disparities in thrombus composition, particularly Semaphorin-7A (Sema7A) and CD163, between patients diagnosed with large-artery atherosclerosis (LAA) and those with CE, and to investigate their potential association with prognosis.

**Methods:**

Thrombi were collected from patients who underwent mechanical thrombectomy at two hospitals. The patients were categorized into two groups: LAA and CE. We compared the levels of Sema7A and CD163 between these groups and analyzed their relationships with stroke severity, hemorrhagic transformation and prognosis.

**Results:**

The study involved a total of 67 patients. Sema7A expression was found to be significantly higher in the CE group compared to LAA (*p* < 0.001). Conversely, no statistically significant differences were observed for CD163 between the groups. The presence of Sema7A/CD163 did not show any associations with stroke severity or hemorrhagic transformation (all *p* > 0.05). However, both Sema7A (OR, 2.017; 95% CI, 1.301–3.518; *p* = 0.005) and CD163 (OR, 2.283; 95% CI, 1.252–5.724; *p* = 0.03) were associated with the poor prognosis for stroke, after adjusting for stroke severity.

**Conclusion:**

This study highlights that CE thrombi exhibited higher levels of Sema7A expression compared to LAA thrombi. Moreover, we found a positive correlation between Sema7A/CD163 levels and the poor prognosis of patients with acute ischemic stroke.

## Introduction

Stroke was a leading cause of mortality worldwide, with ischemic heart disease being the only cause surpassing it, as reported by the Global Burden of Disease Study 2019 [1]. In China, Stroke was also a serious health problem, with increasing morbidity and mortality year by year. According to a study published in the Lancet Public Health, there were 3.94 million new strokes in 2019 alone [2]. The China Stroke High-risk Population Screening and Intervention Program estimated that 17.8 million adults in China had experienced a stroke in 2020. [3].

Acute ischemic stroke (AIS) classification is crucial for determining appropriate treatment strategies and predicting prognosis. Stroke guidelines recommend anticoagulation for cardiogenic stroke and antiplatelet therapy for non-cardiogenic stroke [4]. Furthermore, cardioembolism (CE) cases are associated with a poorer prognosis compared to large-artery atherosclerosis (LAA) cases [5]. To achieve higher clinical benefit, more accurate treatment options and prognostic assessments, it is necessary to help clinicians clarify whether the etiology is CE or non-CE from stroke of undetermined etiology (SUE).

However, there is no consensus on the link between thrombotic components and stroke etiology [6]. Conventional methods for identifying stroke etiology involve analyzing the composition of red blood cells (RBC), fibrous tissue, and platelets within the thrombus. However, these studies have yielded contradictory conclusions. Some studies have reported that patients with LAA or non-CE stroke have a higher proportion of red blood cells in their thrombus [7–9], a higher proportion of fibers [10, 11], and a higher proportion of platelets [12]. Other studies have reached opposite conclusions, finding that patients with LAA stroke have a lower percentage of red blood cells in the thrombus [10, 13], a lower fiber ratio [9, 14], and a lower platelet ratio [9, 15]. These discrepancies suggest that identifying stroke type based on the cellular component content within the thrombus is unreliable.

Therefore, we further investigated the expression of cytokines in thrombus and hypothesized that the expression of specific cytokines may change depending on the origin of the thrombus. Previous studies have demonstrated the association of Semaphorin-7A (Sema7A) with various diseases such as multiple sclerosis lesions [16], rheumatoid arthritis [17], acute aortic dissection [18], etc. It has been reported that CD163 may be a potential biomarker for infectious diseases [19], immune diseases [20], tumors [21] and Neurological Disorders [22]. Sema7A [23] and CD163 [24] have elevated expression in response to inflammation, and they both play a role in atherogenesis. In the case of stroke, serum Sema7A levels have been associated with risk of morbidity [25], whereas serum CD163 has been reported to be negatively associated with prognosis [26, 27].

The relationship between Sema7A/CD163 expression in thrombus components and stroke etiology and prognosis remains unclear. Based on previous studies, we postulated that there are differences in Sema7A/CD163 expression between different stroke subtypes and prognosis. The primary objective of this study was to investigate the variations in Sema7A/CD163 expression in thrombi extracted mechanically from patients with acute ischemic stroke due to LAA or CE. Additionally, we sought to analyze the correlation between Sema7A/CD163 expression and functional prognosis as well as hemorrhagic transformation.

## Methods

### Study design and population

This study was a multicenter retrospective study conducted at the First Affiliated Hospital of Soochow University from August 2019 to August 2022 and the Second People’s Hospital of Lianyungang from January 2021 to July 2021.

Patients eligible for thrombolysis were selected based on the criteria recommended by the Chinese Guidelines for Endovascular Treatment of Acute Ischemic Stroke 2018 [28]. Inclusion criteria were: (1) age ≥ 18 years; (2) presence of acute ischemic lesions in the anterior circulation, confirmed by imaging methods (magnetic resonance imaging or computed tomography); (3) received endovascular treatment. Exclusion criteria were: (1) TOAST-defined subtypes as Small-artery occlusion, Stroke of other determined etiology, or SUE; (2) thrombotic specimens unsuitable for histological analysis; (3) incomplete clinical information.

### Data collection

Data on baseline characteristics, including age, gender, systolic blood pressure (SBP), diastolic blood pressure (DBP), hypertension (HPT), diabetes mellitus (DM), smoking history, thrombolytic therapy, glucose levels (Glu), hemorrhagic transformation,TOAST classification, National Institutes of Health Stroke Scale (NIHSS) scores, and modified Rankin scores (mRS) were collected. Blood sugar was tested on admission.

Subtypes of stroke were classified upon admission by a trained neurologist using TOAST criteria based on clinical and radiological data [29]. LAA: Diagnosis was based on clinical and imaging evidence of significant (> 50%) stenosis or occlusion of the large cerebral arteries. CE: Diagnosis was based on the presence of atrial fibrillation, post-infarction left ventricular wall motion retardation, dilated cardiomyopathy, or other cardiac conditions prone to thrombus formation and/or stroke, as observed on imaging.

Hemorrhagic transformation was defined as a first CT/MRI after cerebral infarction that did not reveal hemorrhage and a second cranial CT/MRI that revealed intracranial hemorrhage.

Function outcomes of AIS were evaluated using the mRS at 90-day discharge. Scores of 0 to 2 were defined as excellent functional outcomes, while scores of 3 to 6 were classified as poor. The mRS score was assessed by medical professionals using a telephone follow-up 3 months after discharge from the hospital.

### Endovascular procedures

**Fig. 1:**
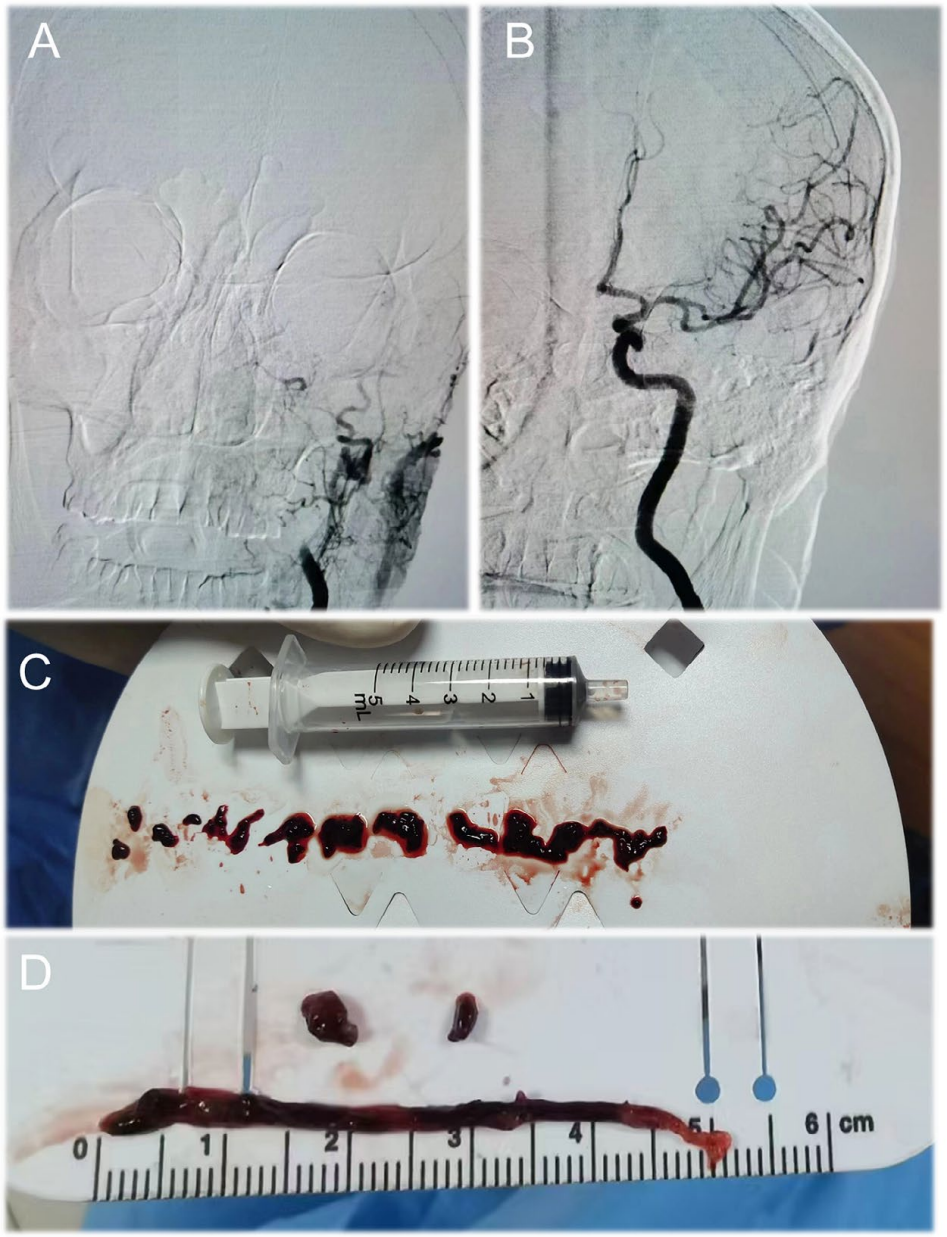
Panel **A** shows cerebral angiography, where the patient’s internal carotid artery was not visualized. Panel **B** illustrates the restoration of blood flow after embolization treatment. Panels **C** and **D** depict emboli of cardiogenic stroke and large artery type, respectively

The surgical procedures for endovascular operations adhered to the guidelines [28]. The treatment strategy for acute ischemic stroke was determined by the treating surgeon, which included catheter aspiration, stent retrieval, or a combination of both techniques. Thrombus samples were collected from patients at the end of the procedure for use in the study (Fig. 1).

### Immunohistochemical staining

**Fig. 2:**
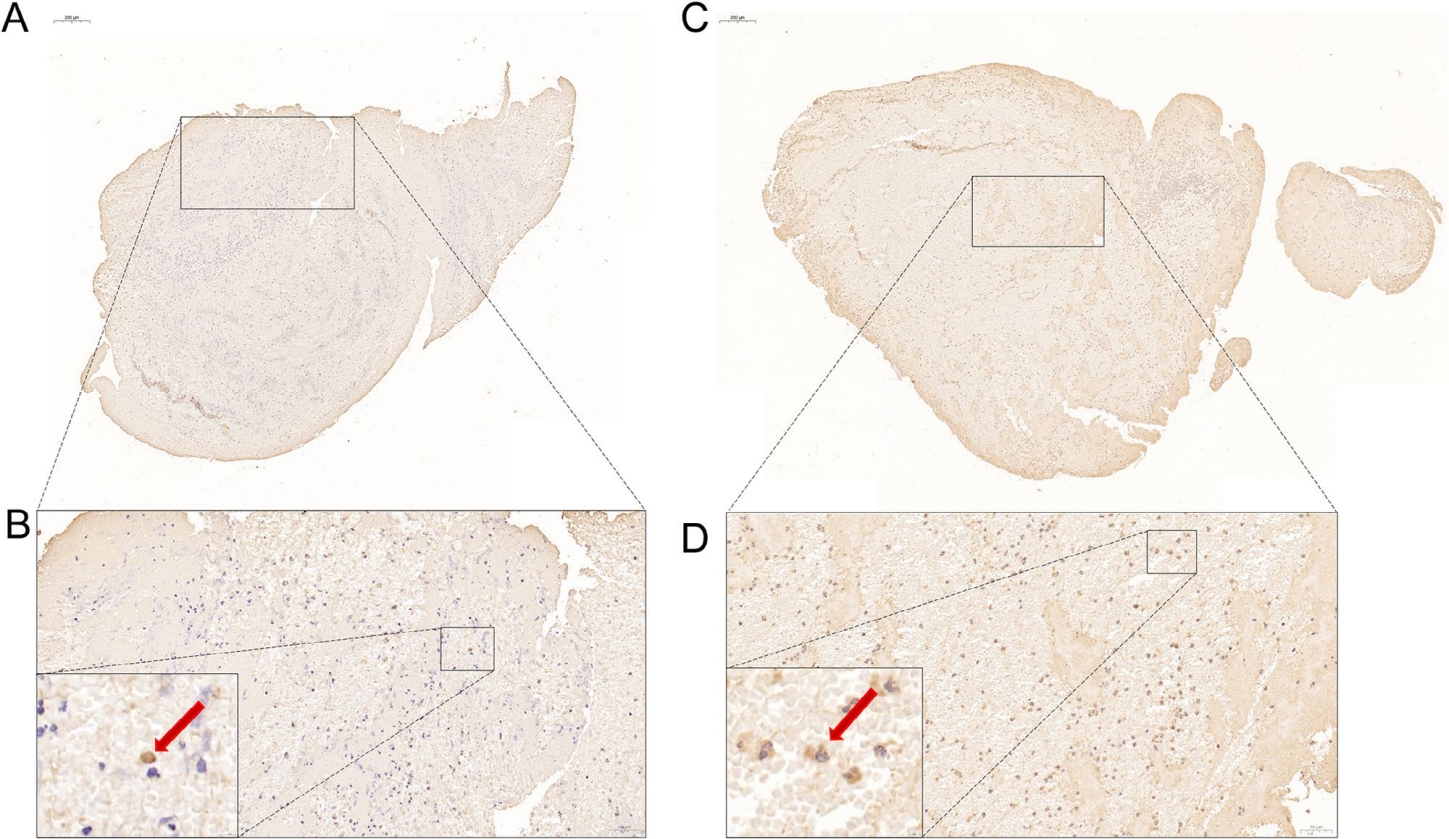
Figure **A** and **B** display Sema7A, while Figure **C** and **D** shows CD163. Figure **A** and **C** together represent the full microscopic field of view from a random section. Figure **B** and **D** indicate the field of view used by the pathologist for counting, with red arrows pointing out the positive macrophages

Retrieved thrombi were promptly fixed in 10% neutral buffered formalin, embedded in paraffin, and sectioned into 4-µm-thick slices. Immunohistochemistry was performed on a randomly selected section using primary antibodies targeting Sema7A (Servicebio, GB111988, 1:500) and CD163 (Servicebio, GB113751, 1:500). Two blinded pathologists, Zhichao Sun and Lei Xu, conducted the staining and analysis. Photomicrographs were obtained at 200x magnification using an Eclipse Ci-L microscope. The two most abundant fields of view with positive macrophages were selected for each section, and their mean value was recorded as one reading result (Fig. 2). The final count of positive cells was calculated as the mean of the pathologists’ results. The ratio of positive macrophages to total macrophages in all four observed fields of view was determined.

### Statistical analysis

Baseline characteristics were presented as mean ± standard deviation (SD), median (range), or frequency and proportion. T-test (for normal continuous variables), Fisher’s exact test (for categorical variables), and Wilcoxon signed-rank test (for non-normal continuous variables) were employed to compare baseline characteristics, treatment data, and functional outcomes in patients with different types of AIS. Spearman correlation analysis was used to assess the relationship between NIHSS and Sema7A/CD163 levels. The study also analyzed the differences in Sema7A/CD163 expression between hemorrhagic transformation and functional prognosis. Univariate analysis was conducted to examine the association between thrombotic composition and functional outcome. Variables that showed significance in the univariate analysis were included in the multifactorial logistic regression for further analysis. All statistical analyses were performed using R 4.2.3 software, with p-values < 0.05 considered statistically significant.

## Results

### Baseline clinical characteristics

**Fig. 3:**
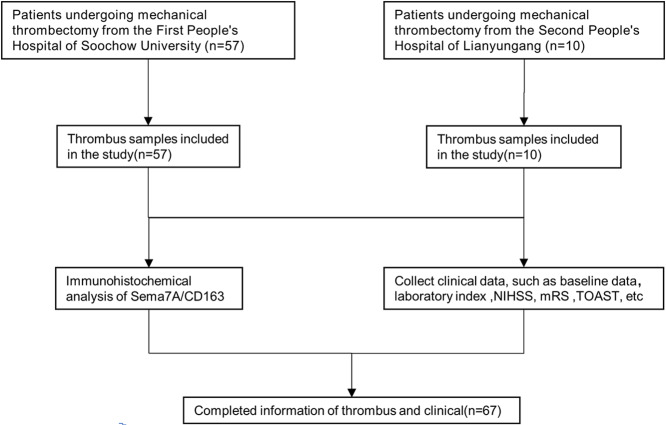
The flow chart

**Fig. 4:**
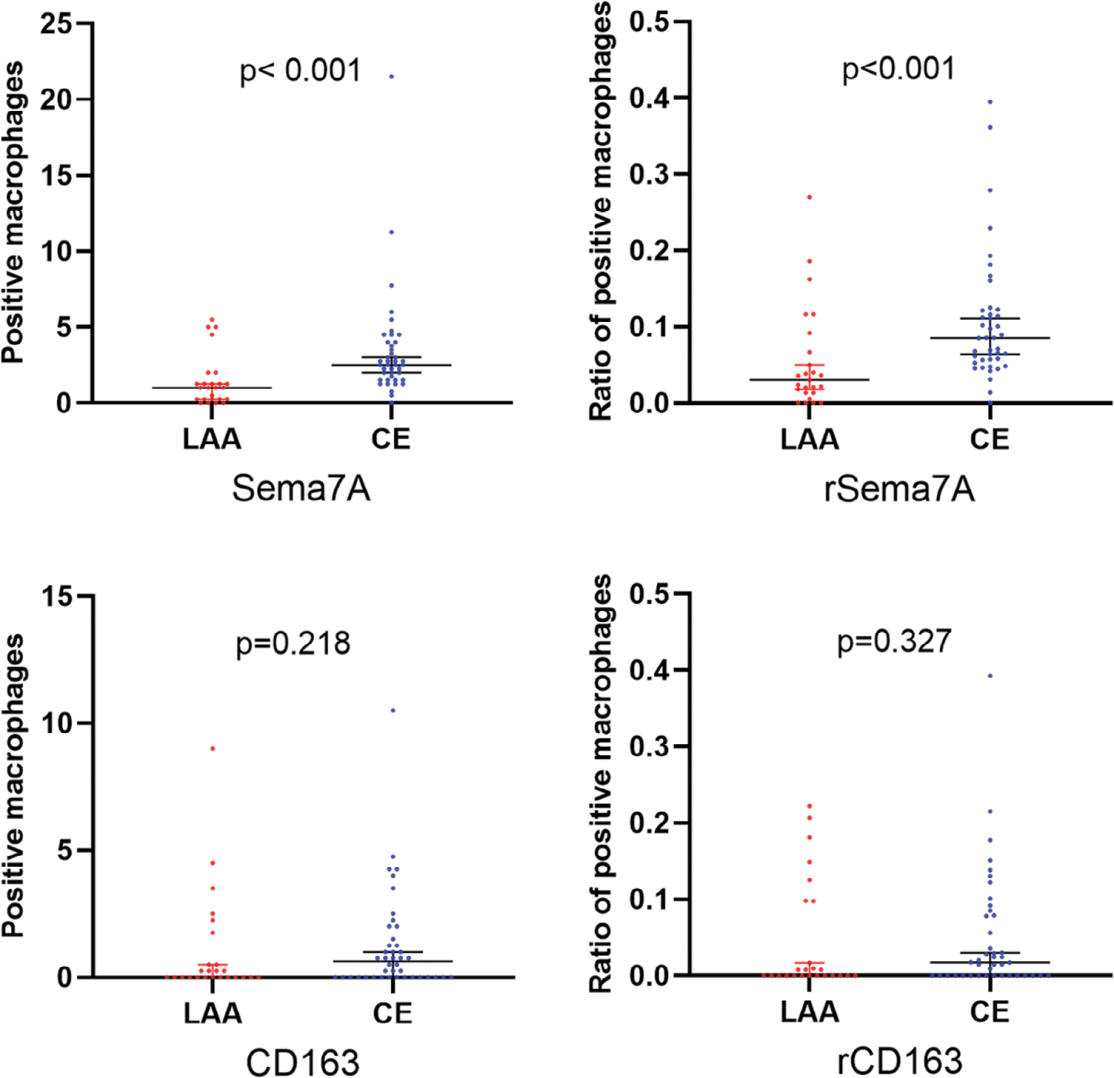
Sema7A expression was significantly higher in cardioembolism (CE) compared to other groups, with both higher numbers and a higher ratio. However, there were no significant between-group differences for CD163 and its ratio

We collected a total of 67 thrombus samples. The flow chart is shown in Fig. 3. Among the participants, 10 cases were from the Second People’s Hospital of Lianyungang, and 57 cases were from the First People’s Hospital of Soochow University. The baseline characteristics of the patients are presented in Table 1. Out of these, 45 patients were diagnosed with CE, while 22 patients had LAA. Significantly higher expression of Sema7A was observed in the CE group, both in terms of absolute numbers and ratios. However, there were no significant differences between the two groups concerning CD163 expression or other variables. The distribution of Sema7A and CD163 between LAA and CE is illustrated in Fig. 4.

**Table 1:** Clinical characteristics

Variables	Total (*n* = 67)	CE (*n* = 42)	LAA (*n* = 25)	Statistic	*P*
Age, Median (Q1, Q3)	68 (59, 75)	72.5 (59.5, 77)	68 (60, 73)	601	0.327
Gender, n (%)				0.658	0.417
Female	27 (40)	19 (45)	8 (32)		
Male	40 (60)	23 (55)	17 (68)		
Systolic blood pressure, Mean ± SD	152.04 ± 24.8	152.76 ± 23.52	150.84 ± 27.28	0.293	0.771
Diastolic blood pressure, Mean ± SD	85.4 ± 14.3	86.07 ± 12.52	84.28 ± 17.11	0.456	0.651
Glucose, Median (Q1, Q3)	6.7 (6.27, 8.09)	6.68 (6.18, 7.79)	6.85 (6.5, 8.25)	450	0.334
NHISS, Mean ± SD	15.22 ± 7.02	15.45 ± 7.37	14.84 ± 6.53	0.354	0.725
Hypertension, n (%)	41 (61)	22 (52)	19 (76)	2.754	0.097
Diabetes mellitus, n (%)	10 (15)	4 (10)	6 (24)	Fisher	0.157
Smoking, n (%)	19 (28)	8 (19)	11 (44)	3.653	0.056
Treatment, n (%)				0.008	0.929
no-rtPA	42 (63)	27 (64)	15 (60)		
rtPA	25 (37)	15 (36)	10 (40)		
90 day-mRS, n (%)				1.519	0.218
0–2	22 (33)	11 (26)	11 (44)		
3–6	45 (67)	31 (74)	14 (56)		
Sema7A, Median (Q1, Q3)	2 (1.12, 3.38)	2.5 (1.56, 3.94)	1 (0.25, 1.25)	831.5	< 0.001
rSema7A, Median (Q1, Q3)	0.06 (0.04, 0.11)	0.09 (0.06, 0.12)	0.03 (0.01, 0.07)	798	< 0.001
CD163, Median (Q1, Q3)	0.25 (0, 1.38)	0.62 (0, 1.44)	0 (0, 0.5)	616.5	0.218
rCD163, Median (Q1, Q3)	0.01 (0, 0.08)	0.02 (0, 0.08)	0 (0, 0.1)	598	0.327

*Abbreviation* large-artery atherosclerosis (LAA), cardioembolism (CE), the National Institutes of Health Stroke Scale (NIHSS), recombinant tissue plasminogen activator (rt-PA, the modified Rankin score (mRS) and semaphorin-7A (Sema7A). The macrophage ratios (the number of positive macrophages divide the number of all macrophages) regarding Sema7A and CD163 were abbreviated as rSema7A and rCD163

### Correlation of Sema7A/CD163 with clinical characters

**Fig. 5:**
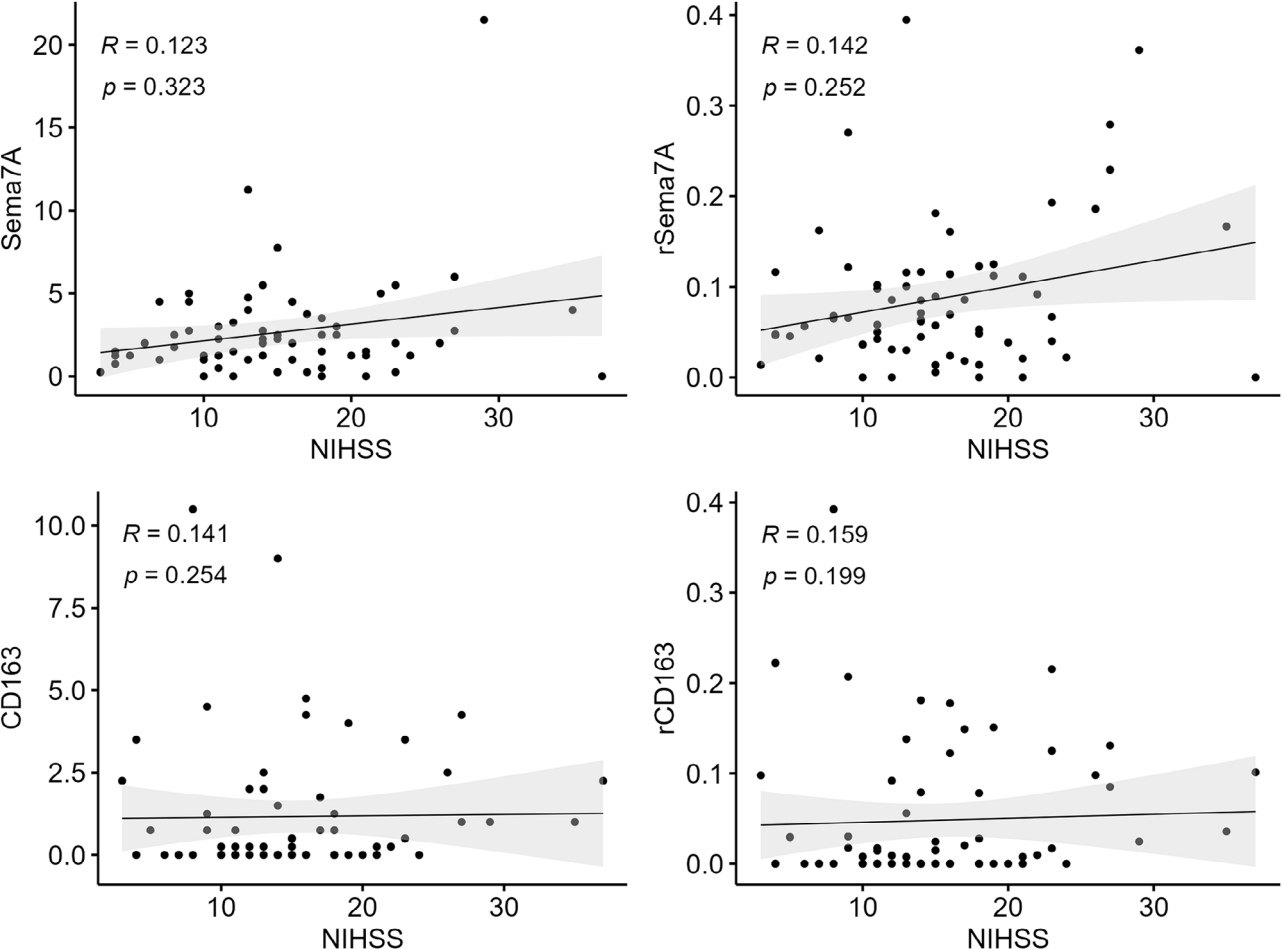
The National Institutes of Health Stroke Scale (NIHSS) did not correlate with counts and ratios of Sema7A and CD163

Regarding the correlation between Sema7A/CD163 and clinical characteristics, stroke severity assessed by NIHSS did not show any significant correlation with the counts and ratios of Sema7A/CD163 (Fig. 5).

Out of the total cases, 15 experienced postoperative hemorrhagic transformation, while the remaining 52 cases did not. However, the expression levels of Sema7A and CD163 were not significantly different between the two groups (Table 2).

**Table 2:** The relationship between hemorrhagic transformation and Sema7A/CD163

Hemorrhagic Transformation	No (*n* = 52)		Yes(*n* = 15)	*P*	
Sema7A, Median (Q1, Q3)	2.25 (1.25, 3.81)	1.5 (1, 2.25)			0.291
rSema7A, Median (Q1, Q3)	0.07 (0.04, 0.12)	0.04 (0.03, 0.09)			0.262
CD163, Median (Q1, Q3)	0.38 (0, 2)	0.25 (0, 0.62)			0.319
rCD163, Median (Q1, Q3)	0.02 (0, 0.1)	0.01 (0, 0.02)			0.249

### Univariate analysis of clinical characters and Sema7A/CD163 between prognosis

The univariate logistic analysis revealed that NIHSS (*P* = 0.01) and Sema7A/CD163 (all *P* < 0.05) were associated with the prognosis. A multifactorial logistic regression analysis, with NIHSS included, demonstrated that after adjusting for NIHSS, Sema7A (OR, 2.017; 95% CI, 1.301–3.518; *P* = 0.005), rSema7A (OR, 2.23*10^12^; 95% CI, 1.999*10^5^-2.864*10^21^; *P* = 0.003), CD163 (OR, 2.283; 95% CI, 1.252–5.724; *P* = 0.03), and rCD163 (OR, 1.74*10^6^; 95% CI, 36.790–4.373*10^13^; *P* = 0.03) remained statistically significant (Table 3).

**Table 3:** Regression analysis about stroke prognosis

	Univariate regression				Multifactor regression		
Characteristics	OR	CI	P		OR	CI	*P*
Age	1.035	0.991–1.08	0.12				
Gender	1.373	0.488–3.857	0.55				
Systolic blood pressure	1.007	0.986–1.029	0.51				
Diastolic blood pressure	1.01	0.975–1.047	0.57				
Glucose	1.002	0.765–1.313	0.99				
NHISS	1.139	1.032–1.258	0.01				
TOAST	0.452	0.158–1.287	0.14				
Hypertension	1.51	0.535–4.264	0.44				
Diabetes mellitus	2.162	0.418–11.171	0.36				
Smoking	1.535	0.472–4.997	0.48				
Treatment	1.062	0.369–3.058	0.91				
Hemorrhagic transformation	1.456	0.405–5.231	0.56				
Sema7A	1.935	1.231–3.04	0.004		2.017	1.301–3.518	0.005
rSema7A	2.23E + 10	4765.544-1.044E + 17	0.002		2.23E + 12	1.999e + 05-2.864E + 21	0.003
CD163	2.315	1.097–4.888	0.03		2.283	1.252–5.724	0.03
rCD163	7.39E + 06	6.737-8.112E + 12	0.03		1.74E + 06	36.790-4.373E + 13	0.03

*Abbreviation* Semaphorin-7A (Sema7A), the trial of Org 10,172 in Acute Stroke Treatment (TOAST), the National Institutes of Health Stroke Scale (NIHSS), The macrophage ratios (the number of positive macrophages divide the number of all macrophages) regarding Sema7A and CD163 were abbreviated as rSema7A and rCD163. Treatment means patients with thrombolytic therapy or not. E + means the number of decimal places

**Fig. 6:**
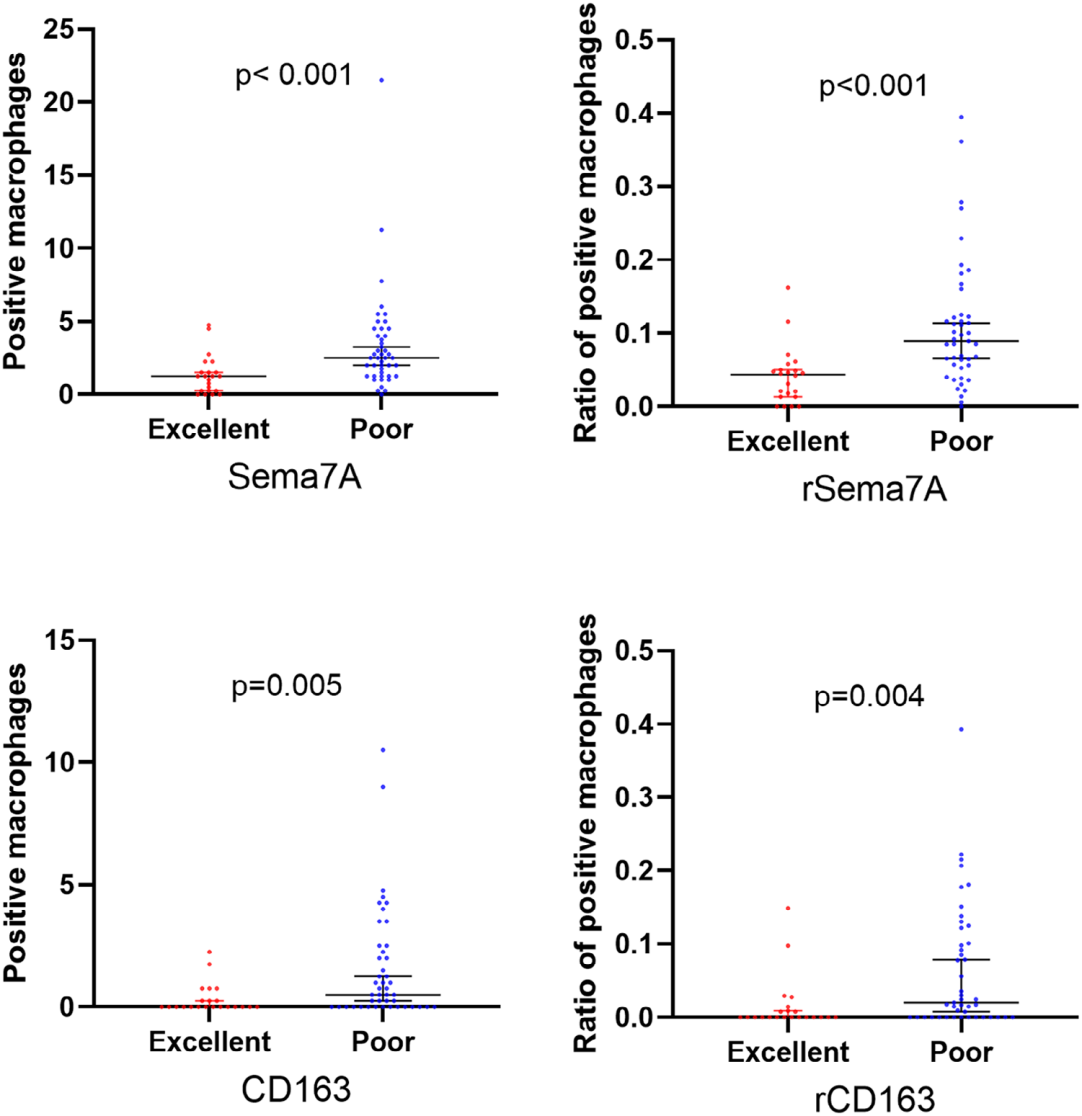
The relationship between the prognosis of stroke and Sema7A/CD163. The expression of Sema7A and CD163 was higher in the poor prognosis group

Regarding the prognosis at 90 days, 22 cases demonstrated an excellent outcome, while 45 cases had a poor prognosis. The poor prognosis group exhibited higher levels of Sema7A and CD163 expression. Sema7A (*P* < 0.001), rSema7A (*P* < 0.001), CD163 (*P* = 0.005), and rCD163 (*P* = 0.004) were all statistically significant (Fig. 6).

## Discussion

In this study, we investigated the relationship between Sema7A/CD163 expression in thrombi and stroke classification, along with other clinical characteristics. Our findings suggest that Sema7A expression has discriminatory significance in distinguishing between LAA and CE. Additionally, we explored the association of Sema7A/CD163 expression with the prognosis of AIS.

Thrombus formation is a complex interplay of various immuno-inflammatory molecules and signaling pathways [30–35]. As a result, certain immunoinflammatory mediators show promise for identifying the source of thrombus. For example, LAA thrombi have been found to contain a higher proportion of CD3 + T cells compared to CE thrombi, suggesting that CD3 + T cells may serve as a reliable biomarker for early stage LAA thrombosis [36]. Another study by Wang et al. demonstrated the presence of more actin and CD105 + endothelial cells in LAA compared to CE cases [37].

CD163, a hemoglobin-binding protein receptor present on macrophages, it high express in macrophages is a feature of tissue response to inflammation [38]. CD163 expression is upregulated in a variety of inflammatory and malignant diseases and is altered by induction from inflammatory factors such as IL, TNF, IFN and LPS [39]. Guo et al. reported that CD163 + macrophages were linked to plaque progression, microangiogenesis, and elevated levels of hypoxia-inducible factor 1α (HIF-1α) and vascular endothelial growth factor-a expression [24]. Furthermore, iron deprivation-induced inhibition of prolyl hydroxylase function in CD163 + macrophages may trigger HIF-1α-mediated angiogenesis, increased vascular permeability, and exacerbation of microvascular inflammation—features commonly observed in advanced atherosclerotic plaques. This suggests that CD163 + macrophages accelerate plaque destabilization through an inflammatory response, culminating in intraplaque hemorrhage, inflammatory cell recruitment, enlargement of the necrotic core, plaque rupture, and LAA thrombosis [24].

In our study, CD163 in LAA and CE did not demonstrate differences. We hypothesize that CD163 is involved in the process of thrombosis whatever in aortic thrombosis and cardiogenic thrombosis because of its extensive participation in various inflammatory responses.

Sema7A is a molecule expressed in immune cells, such as T lymphocytes, monocytes, dendritic cells, and platelets. It plays a crucial role in regulating various immune inflammatory processes, including immune cell interactions, inflammatory infiltration, and cytokine production. Additionally, Sema7A acts as an endogenous mediator, which is essential for both inducing and resolving the acute inflammatory response, thereby contributing to host defense and tissue homeostasis [40]. There is a lack of research on Sema7A and stroke etiology. Shuhong Hu et al. established a mouse model of disturbed blood flow (d-flow) by a partial carotid artery ligation and found that upregulation of Sema7A in large arteries during d-flow led to endothelial dysfunction via endothelial integrin β1 [23]. A possible downstream pathway is the subsequent upregulation of the transcription factor ATF3 by integrin β1, leading to enhanced TGF-β2 transcription and activation of the TGF/Smad3 signaling pathway, which induces endothelial-to-mesenchymal transition [41]. David et al. found that Sema7A released from erythrocyte injury promotes thrombosis and thrombotic myocardial injury by interacting with glycoprotein Ib in the presence of hemodynamic disturbances [42]. Based on these studies we try to explain the results of our study. It is well known that common risk factors for cardioembolism are atrial fibrillation and rheumatic heart disease, which imply severe hemodynamic disturbances. Therefore, we hypothesized that due to the more severe hemodynamic disturbances of CE than LAA. In CE, upregulation of Sema7A interaction with endothelial integrin β1 or glycoprotein Ib mediates downstream signaling pathways to form a thrombus inducing cardioembolic stroke. This seems to explain the higher expression of Sema7A compared with LAA in CE thrombi in our study.

Multiple immunoinflammatory molecules present in thrombi may provide insight into stroke regression. Gong et al. discovered a negative association between that intra-thrombotic CD4+/CD25 + T cell levels and hemorrhagic transformation after mechanical thrombectomy (MT) [43]. Similarly, Mereuta et al. using CD 34 to label endothelial cells, found that MT may be linked to vascular wall injury, resulting in poor regression [44]. In contrast, Boeckh-Behrens et al. demonstrated that AIS patients with an enrichment of CD31 + cells within the thrombus had better early regression, and CD31 was suggested as a potential neuroprotective agent for stroke patients [45].

In our study, we observed a positive association of Sema7A/CD163 in thrombosis with poor prognosis of AIS. However, the precise underlying mechanism of action remains unclear. Only one study has described a relationship between Sema7A and stroke. The study involving 105 patients with LAA and 105 healthy controls, reported the LAA serum Sema7A levels was three times than the controls [25]. Additionally, a study of 378 subjects showed a negative association between CD163 in serum and stroke prognosis [26]. This is inconsistent with our result. Due to the difference in the source of CD163 expression, its study sample was serum and our study sample was thrombus. We are not sure whether the expression of CD163 in serum and thrombus is consistent. This also points out the direction for our next research. The above studies collectively suggest that Sema7A/CD163 serves as a predictive factor for poor stroke prognosis, both in serum and thrombus. In short, Sema7A/CD163 has potential as a prognostic marker for stroke, but more rigorous studies are still needed to validate the conclusions.

Numerous studies have demonstrated the relationship between Sema7A/CD163 and the progression of arterial plaques, as well as their impact on plaque vulnerability. Sema7A promotes neovascularization in a β1 integrin-dependent manner, making arterial plaques more susceptible to rupture [46]. Although the role of CD163 in stroke arterial plaques remains to be fully understood, HU et al. examined 200 carotid plaques obtained during carotid endarterectomy and observed an association between CD163 expression and increased plaque vulnerability [47]. Atsushi et al. reported that CD163 macrophages inhibit vascular calcification [48]. Additionally, experiments in mice have shown that CD163 macrophages decelerate the development of atherosclerotic plaques [49]. In our study, neither NIHSS nor hemorrhagic transformation showed significant associations with Sema7A/CD163. The nature of the thrombus may influence the ease of the embolization procedure, with hard thrombi posing greater challenges and fragile thrombi being prone to distal embolization or secondary hemorrhagic transformation. However, the correlation between dislodged thrombus and arterial plaque remains a randomized, risky event and requires further exploration.

This study had several limitations that should be acknowledged. Firstly, the data used for analysis were obtained from two views of one section of a thrombus, which may not fully represent the entire thrombus composition. Secondly, due to the retrospective nature of the trial, we were unable to collect patients’ blood samples and other relevant clinical information, such as the number of surgeries and the duration of surgery. Thirdly, Sema7A is widely present in various cells, but in this study, we only counted the number of positively stained macrophages. Lastly, the sample size was relatively small, necessitating caution in interpreting the results of the data analysis.

In general, current studies on Sema7A/CD163 and stroke are mainly based on serum and arterial components. Our study discusses the relationship between Sema7A/CD163 and stroke etiology and prognosis based on thrombus samples. Our next plan is to refine the collection of serum samples and thrombus samples to explore the consistency of Sema7A and CD163 expression in thrombus and serum samples. Further searching for potential markers for intervention in the stroke process

## Conclusion

Pathological analysis of thrombus components obtained through MT plays a crucial role in determining stroke etiology and pathogenesis. Additionally, it can aid in stroke type differentiation and guide prognosis. In our study, we observed higher expression of Sema7A in thrombi from patients with CE compared to LAA. Moreover, Sema7A/CD163 expression was positively associated with the poor prognosis.

## Data Availability

The datasets used and/or analysed during the current study are available from the corresponding author on reasonable request.

## Abbreviations

Sema7A: Semaphorin-7a
LAA: Large-artery atherosclerosis
CE: Cardioembolism
AIS: Acute ischemic stroke
TOAST: The Trial of Org 10172 in acute stroke treatment
SUE: Stroke of undetermined etiology
HIF-1α: Hypoxia-inducible factor 1α
SBP: Systolic blood pressure
DBP: Diastolic blood pressure
HPT: Hypertension
DM: Diabetes mellitus
Glu: Glucose levels
NIHSS: National Institutes of Health Stroke Scale
mRS: Modified Rankin scores
SD: Standard deviation
MT: Mechanical thrombectomy
